# Laparoscopic Surgery via an Inferior Pancreatic Border Approach for Median Arcuate Ligament Syndrome after Distal Gastrectomy: A Case Report

**DOI:** 10.70352/scrj.cr.25-0173

**Published:** 2025-06-10

**Authors:** Kazuki Nishino, Mizuki Ninomiya, Naotaka Kuriyama, Takuma Izumi, Shuhei Kajiwara, Ryota Nakanishi, Yoshihiko Fujinaka, Shigeyuki Nagata, Hiroto Kayashima

**Affiliations:** Department of Surgery, Aso Iizuka Hospital, Iizuka, Fukuoka, Japan

**Keywords:** median arcuate ligament syndrome, inferior pancreatic border approach, laparoscopic ligament division

## Abstract

**INTRODUCTION:**

Median arcuate ligament syndrome (MALS) is a relatively rare condition characterized by compression of the celiac artery and neural plexus by the median arcuate ligament. The definitive treatment is surgical division of the median arcuate ligament, which can be performed via open, laparoscopic, or retroperitoneoscopic surgery. In laparoscopic surgery, this approach commonly involves reaching the root of the celiac artery from the superior border of the pancreas to divide the median arcuate ligament. We herein report a case of MALS following gastrectomy, in which the median arcuate ligament was successfully divided using a laparoscopic approach from the inferior border of the pancreas.

**CASE PRESENTATION:**

A 67-year-old man who had undergone open distal gastrectomy and D2 lymph node dissection for gastric cancer 16 years prior presented to the emergency department with a chief complaint of sudden, severe epigastric pain. A thorough examination led to the diagnosis of acute cholangitis and pancreatitis due to retroperitoneal hematoma compression, for which percutaneous transhepatic cholangial drainage (PTCD) was performed. Additionally, MALS was identified and considered as the underlying cause of retroperitoneal hematoma. Conservative treatment resulted in hematoma shrinkage and improvement in cholangitis and pancreatitis, allowing the patient to be discharged. However, because of the possibility of symptom recurrence, the patient was referred to our department for surgical intervention. Given the history of gastrectomy, an approach from the superior border of the pancreas was deemed to be challenging. Instead, we accessed the root of the celiac artery from the inferior border of the pancreas, successfully dividing the median arcuate ligament and confirming the improved blood flow.

**CONCLUSIONS:**

MALS is a rare condition for which a standardized surgical approach remains to be established. In cases of adhesions in the upper abdomen, an approach from the inferior border of the pancreas may be a viable option and could contribute to improved surgical outcomes.

## Abbreviations

CT
: computed tomography
IMV
: inferior mesenteric vein
MALS
: median arcuate ligament syndrome
PTCD
: percutaneous transhepatic cholangial drainage
RAMPS
: radical antegrade modular pancreatosplenectomy
SMA
: superior mesenteric artery

## INTRODUCTION

The median arcuate ligament is a fibrous band that forms the anterior margin of the aortic hiatus, and connects the right and left crura of the diaphragm. MALS is a relatively rare condition characterized by compression of the celiac artery and neural plexus by the median arcuate ligament.^1)^ The symptoms include postprandial abdominal pain, weight loss, abdominal vascular murmurs, nausea, and vomiting.^2)^ Additionally, prolonged hemodynamic stress can lead to aneurysm formation, resulting in hemorrhage or hematoma.^3)^ The definitive treatment for MALS involves surgical division of the median arcuate ligament. Reported surgical approaches include open surgery, as well as laparoscopic and retroperitoneoscopic approaches.^4,5)^ The conventional laparoscopic approach involves accessing the origin of the celiac artery from the superior border of the pancreas to perform ligament division.^6,7)^ However, when a patient has a history of upper abdominal surgery, adhesions around the pancreas make it difficult to approach via the standard route. One established surgical approach for pancreatic cancer in distal pancreas is the RAMPS procedure, which involves accessing the pancreas from its inferior margin. This method is considered effective in cases with postoperative adhesions following gastric cancer surgery, as it allows for a safer and more controlled dissection through a familiar anatomical route, reducing the risk of complications associated with dense adhesions.

We herein report a case in which laparoscopic surgery was performed via an inferior pancreatic border approach to access the origin of the celiac artery, as the superior border approach was challenging due to a history of distal gastrectomy.

## CASE PRESENTATION

**Fig. 1:**
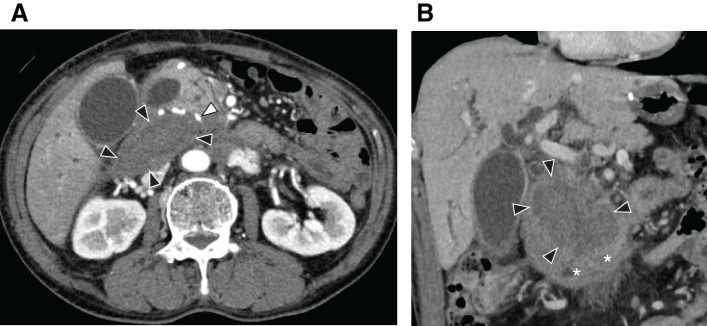
Computed tomography findings of the patient upon presentation: (**A**) axial and (**B**) coronal sections of the computed tomography scans showed 70 × 50 mm hypodense hematoma (black arrowheads) on the posterior aspect of the pancreatic head. The white arrowhead indicates the dilated inferior pancreatoduodenal artery.

**Fig. 2:**
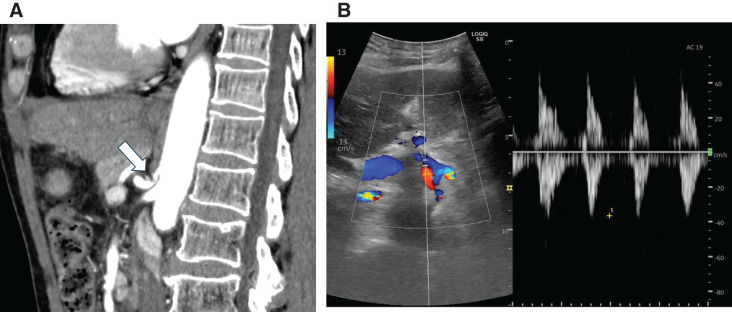
(A) Computed tomography (sagittal section) revealed stenosis at the origin of the celiac artery (white arrow). (**B**) A Doppler ultrasound examination revealed retrograde blood flow in both the gastroduodenal artery and the common hepatic artery. The common hepatic artery was retrograde and had a velocity of 36.2 cm/s.

A 67-year-old male patient presented to the emergency department with chief complaints of anorexia and fatigue. Two weeks prior to presenting to our hospital, he had experienced sudden-onset upper abdominal pain, which subsided within a few days. He had a medical history of open distal gastrectomy and D2 lymph node dissection with Roux-en-Y reconstruction for gastric cancer 16 years prior. Upon examination, mild epigastric tenderness and yellowing of the conjunctiva and skin were observed. Blood tests revealed elevated levels of biliary enzymes, pancreatic enzymes, and markers of liver inflammation. Abdominal contrast-enhanced CT at the end of inhalation showed a 70 × 50 mm hypodense, but relatively high-density fluid collection compared with bile fluid on the posterior aspect of the pancreatic head. It also showed a dilated intrahepatic bile duct and common bile duct, probably due to compression of the 3rd portion of the duodenum by the hematoma (**Fig. 1A**, **1B**). Additionally, stenosis of the celiac artery origin and dilated inferior pancreatoduodenal arteries, which were located just adjacent to the above fluid collection, were noted (**Fig. 2A**), leading to the diagnosis of retroperitoneal hematoma caused by median arcuate ligament syndrome and subsequent obstructive cholangitis, and pancreatitis due to hematoma compression of the outlets of distal bile duct and pancreatic duct. As no active bleeding or aneurysm formation was observed on CT, probably due to thrombosis after rupture of pre-existed aneurysm, endovascular treatment was not performed, and PTCD was performed for jaundice reduction. Conservative treatment resulted in hematoma reduction and improvement of the patient’s cholangitis and pancreatitis, allowing him to be discharged on the 25th day. Follow-up abdominal ultrasound Doppler examination revealed a mixture of antegrade and retrograde blood flow in the celiac artery. The common hepatic artery was retrograde and had a velocity of 36.2 cm/s (**Fig. 2B**). Based on these findings, a diagnosis of median arcuate ligament syndrome was made. Furthermore, no obvious aneurysm was detected on the CT scan after resolution of the hematoma. Given the possibility of symptom recurrence, the patient was referred to our department for further surgical intervention.

**Fig. 3:**
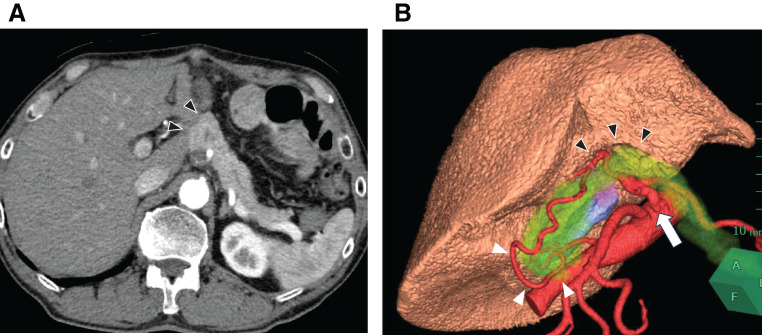
(**A**) Computed tomography demonstrating dense adhesion between the Spiegel lobe of the liver and the pancreas (black arrowheads). (**B**) 3D computed tomography demonstrating stenosis at the origin of the celiac artery (white arrow) and the development of an arterial arcade in the pancreatic head (white arrowheads), dense adhesion between the Spiegel lobe of the liver, and the pancreas (black arrowheads). The translucent yellow represents the pancreatic parenchyma.

As CT images indicated dense adhesion between the Spiegel lobe of the liver and pancreas due to lymph node dissection for gastric cancer, an approach from the superior border of the pancreas was anticipated to be difficult, and an inferior pancreatic border approach was planned (**Fig. 3**).

**Fig. 4:**
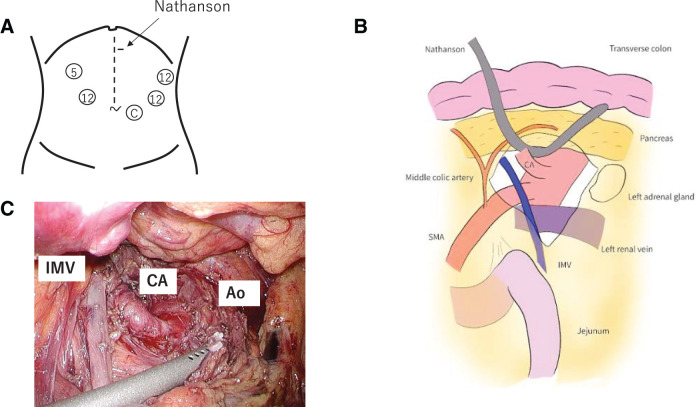
Intraoperative image: (**A**) Trocar placement. The operator stands on the left side of the patient, while the assistant is on the right side and the scopist is between the legs. The Nathanson retractor is placed at the epigastric lesion. C, camera port; 5, 5 mm trocar; 12, 12 mm trocar. (**B**) Schematic illustration of the surgical approach. The transverse mesocolon was opened along the left edge of the inferior mesenteric vein. Dissection was continued along the anterior aspect of Gerota’s fascia, while the transverse colon and pancreas were lifted up using a Nathanson retractor. (**C**) The median arcuate ligament was dissected via the approach from the inferior border of the pancreas. Ao, aorta; CA, celiac artery; IMV, inferior mesenteric vein

Under general anesthesia, the surgery was performed in a supine position with the legs apart. Operator’s specialty was a hepato-biliary-pancreatic surgery. He was also a board-certified surgeon of endoscopic surgical skill qualification system in Japan. A 12-mm balloon port was inserted via an open technique in the left lower abdomen and used as a camera port. Pneumoperitoneum was established at 8 mmHg, and observation of the abdominal cavity revealed extensive adhesions along the previous surgical scar, with adherence of the abdominal wall and mesentery in the upper abdomen. Four additional working ports were placed in the upper abdomen, as shown in **Fig. 4A**. The adhesions were carefully dissected. The stomach remnant and pancreas were found to be adherent to the liver surface, making the approach from the superior pancreatic border difficult. Therefore, an inferior pancreatic border approach was planned starting from the caudal side of the transverse mesocolon. The transverse colon was flipped upward and the jejunal limb and IMV were identified. The transverse mesocolon was incised along the left edge of the IMV. Dissection was continued exposing the anterior aspect of Gerota’s fascia, progressing toward the cranial direction of the retroperitoneum (**Fig. 4B**). The transverse colon and pancreas were lifted using a Nathanson Hook Liver Retractor (Yufu ITONAGA Ltd., Tokyo, Japan). There were no apparent effects of hematoma or post-gastrectomy adhesions around the retroperitoneal space and celiac artery. Intraoperative ultrasonography was used to identify the abdominal aorta, superior mesenteric artery, and celiac artery. Doppler ultrasonography showed no blood flow at the origin of the celiac artery. The neural plexus around the artery was dissected and the celiac artery was exposed, revealing the median arcuate ligament. The median arcuate ligament was divided (**Fig. 4C**), and improvement in celiac artery pulsation and increased blood flow was confirmed using Doppler ultrasound. Hemostasis was confirmed, and a 15 Fr JVAC drain was placed in the posterior pancreas. The pneumoperitoneum was released and the abdominal wall was closed with sutures after ligating the 12-mm port site. The total surgical time was 261 min, with minimal blood loss.

**Fig. 5:**
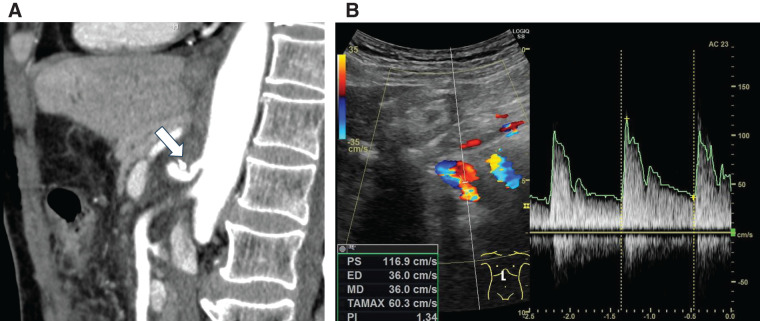
(**A**) Computed tomography (sagittal section) showed that compression of the celiac artery had been relieved (white arrow). (**B**) Postoperative Doppler ultrasound revealed antegrade blood flow in the common hepatic artery with a velocity of 116.9 cm/s.

Oral intake was resumed on the day after surgery. A CT scan on the 4th postoperative day confirmed that compression of the celiac artery was relieved and the absence of pancreatic leaks, allowing for removal of the drain (**Fig. 5A**). No postoperative complications were observed, and the patient was discharged on the 5th postoperative day. Follow-up abdominal Doppler ultrasound in the 1st postoperative month revealed antegrade blood flow in the common hepatic artery with a velocity of 116.9 cm/s (**Fig. 5B**). The patient remained asymptomatic, with no recurrence of symptoms at the 6-month follow-up examination.

## DISCUSSION

MALS is a relatively rare condition, in which the celiac artery is compressed by the median arcuate ligament. It is often found incidentally in imaging studies, with a reported prevalence of 2 per 100000 people.^8)^ With the recent advancements in imaging diagnostics, the number of diagnoses has increased. Symptoms include postprandial abdominal pain, weight loss, nausea, vomiting, and abdominal vascular bruit; however, many patients remain asymptomatic.^2)^ MALS can lead to increased blood flow in the SMA, formation of collateral arterial circulation around the pancreatic head, and the development of visceral artery aneurysms.^9)^

CT imaging is useful for the diagnosis of MALS because it reveals stenosis of the celiac artery.^10)^ Additionally, abdominal Doppler ultrasound can confirm retrograde blood flow in the CHA, which is useful for diagnosis.^11)^ Angiography can be performed if an aneurysm is present. In this case, although the patient exhibited no symptoms other than upper abdominal pain, MALS was diagnosed based on findings from CT imaging and abdominal Doppler ultrasonography.

The primary treatment for MALS is surgical division of the median arcuate ligament, although the indications for surgery have not been clearly defined. Various surgical approaches, including open, laparoscopic, and retroperitoneoscopic surgery, have been reported.^2,4)^ However, owing to the rarity of MALS, there is no standardized surgical method. Laparoscopic approaches commonly involve reaching the celiac artery from the superior pancreatic border. The lesser omentum or omental bursa was opened to identify the celiac artery from the root of the left gastric artery at the superior pancreatic border. The median arcuate ligament is identified at the origin of the celiac artery and divided.^12,13)^ In retroperitoneoscopic surgery, trocars are placed around the left flank portion for a direct approach to the retroperitoneal space. The peritoneum is dissected from the diaphragm downwards until the upper pole of the kidney and supra-renal aorta are visualized.^4)^ Although this approach has some advantages, an approach to the abdominal aorta from the left-posterior side through the retroperitoneal space would be unfamiliar for most abdominal surgeons. In this context, our technique would be more familiar because there are similar surgical technique for pancreatic cancer (RAMPS procedure), dissecting retroperitoneal space through the mesocolon. Intraoperative complications, including arterial injury (6.6%–10%) and pneumothorax (1.9%–2.5%), have been reported.^2,14)^ In addition, the recurrence rate after laparoscopic surgery ranges from 5% to 80%.^2,14,15)^ Although there are numerous reports on laparoscopic surgery for MALS, most describe approaches from the superior border of the pancreas. There have only been two reports from Japan describing an approach from the inferior pancreatic border. In the reported cases, the omental bursa was opened, and the dorsal side of the stomach was elevated to approach the lower margin of the pancreas.

In this case, the patient underwent distal gastrectomy, and the remnant stomach and pancreas showed dense adhesion, making an approach from the superior pancreatic border difficult. In addition, since the patient had undergone Roux-en-Y reconstruction and the elevated jejunum was adhered to the transverse colon, we approached via the transverse mesocolon and elevated the pancreas *en bloc*.

Inappropriate detachment of adhesions around the pancreas may cause pancreatic parenchymal injury. Given the higher risk of aneurysm formation in MALS, pancreatic leaks can potentially lead to aneurysm rupture. Therefore, the approach was modified using a method similar to RAMPS for pancreatic body-tail resection,^16)^ with an approach extending from the inferior pancreatic border to the celiac artery root. This approach provides a clear view of the celiac artery, allowing successful division of the median arcuate ligament.

One limitation of this surgical approach is that, if the surgical team lacks sufficient experience in laparoscopic RAMPS procedure, the complexity of the technique may pose significant technical difficulties. And potential complication associated with this surgical approach is an injury of the left renal artery when unintendedly strayed into the dorsal layer of the Gerota’s fascia in the presence of tortuous anomalies. To prevent this, it is important to confirm the anatomy by preoperative CT or 3D images. These factors must be carefully considered when selecting the appropriate surgical strategy.

In cases with a history of gastric resection, post-inflammatory adhesions, or when the celiac artery originates at the inferior pancreatic border, an approach from the inferior pancreatic border may be a viable option. Future studies with larger sample sizes are necessary to assess surgical outcomes and long-term prognosis.

## CONCLUSIONS

In summary, MALS is a rare condition for which no standardized surgical treatment has been established. In cases involving adhesions in the upper abdomen, particularly near the upper pancreatic border, an approach from the inferior pancreatic border may be a viable option and could lead to improved surgical outcomes.

## ACKNOWLEDGMENTS

We would like to thank Japan Medical Communication for English language editing.

## DECLARATIONS

### Funding

No funding was received for this study.

### Authors’ contributions

KN and MN performed all operations.

KN and MN conceived and designed the study.

The remaining authors (NK, TI, SK, RN, FY, SN) contributed to the collection, analysis, and interpretation of data.

KN prepared the manuscript, and MN critically revised the manuscript.

MN provided the final approval of the version to be published.

All authors have read and approved the final manuscript.

### Availability of data and materials

All data generated during this study are included in this published article.

### Ethics approval and consent to participate

Informed consent was obtained from the patient.

### Consent for publication

Informed consent for the publication of the report and accompanying images was obtained from the patient.

### Competing interests

The authors declare no conflicts of interest in association with the present study.
